# Multilevel Effects of Wealth on Women's Contraceptive Use in Mozambique

**DOI:** 10.1371/journal.pone.0121758

**Published:** 2015-03-18

**Authors:** José G. Dias, Isabel Tiago de Oliveira

**Affiliations:** 1 Instituto Universitário de Lisboa (ISCTE-IUL), BRU—Business Research Unit, Lisboa, Portugal; 2 Instituto Universitário de Lisboa (ISCTE-IUL), CIES—Centro de Estudos e Investigação em Sociologia, Lisboa, Portugal; China Agricultural University, CHINA

## Abstract

**Objective:**

This paper analyzes the impact of wealth on the use of contraception in Mozambique unmixing the contextual effects due to community wealth from the individual effects associated with the women's situation within the community of residence.

**Methods:**

Data from the 2011 Mozambican Demographic and Health Survey on women who are married or living together are analyzed for the entire country and also for the rural and urban areas separately. We used single level and multilevel probit regression models.

**Findings:**

A single level probit regression reveals that region, religion, age, previous fertility, education, and wealth impact contraceptive behavior. The multilevel analysis shows that average community wealth and the women’s relative socioeconomic position within the community have significant positive effects on the use of modern contraceptives. The multilevel framework proved to be necessary in rural settings but not relevant in urban areas. Moreover, the contextual effects due to community wealth are greater in rural than in urban areas and this feature is associated with the higher socioeconomic heterogeneity within the richest communities.

**Conclusion:**

This analysis highlights the need for the studies on contraceptive behavior to specifically address the individual and contextual effects arising from the poverty-wealth dimension in rural and urban areas separately. The inclusion in a particular community of residence is not relevant in urban areas, but it is an important feature in rural areas. Although the women's individual position within the community of residence has a similar effect on contraceptive adoption in rural and urban settings, the impact of community wealth is greater in rural areas and smaller in urban areas.

## Introduction

Wealth, economic growth and income inequality have been associated with health. The overall poverty level and inequality within a country increase health vulnerabilities and decrease life expectancy. Contraceptive adoption is not only a significant component of individual reproductive health, but also an important factor for reducing fertility in developing countries and escaping from the poverty-fertility trap. The association between contraceptive behavior and the poverty-wealth dimension has been studied from the perspectives of equity in maternal health and health coverage gaps. Large scale studies surveying several countries reveal marked differences in the coverage gap in family planning, maternal and child health that is associated with wealth [1–3]. In addition to cross-country analyses with aggregate indexes, studies based on specific health outcomes show significant family planning differentials associated with wealth. For instance, the modern contraceptive rate for the richest women is about double that of the poorest [4–5] and the disparities in the access to family planning, information and direct contact with field workers are associated with the wealth gradient [6]. Furthermore, there is an increasing discrepancy between the absolute poorest and the richest segments in the use of modern contraception [7].

The importance and the inequity of unmet needs for family planning are particularly marked in sub-Saharan Africa [8–9] and specific studies on the African situation confirm the effect of wealth on contraceptive use [10]. Research on how wealth inequalities impact contraceptive adoption tends not to isolate the contextual effects arising from community wealth, although previous studies do show that the characteristics of the community of residence affect both health care utilization and health problems. Nonetheless, research about wealth related contextual effects on the use of modern contraception in Africa is scarce. An examination of six African countries shows that community factors have a marked impact, but the key factors differ between countries [11]. A comprehensive analysis of 21 African countries confirmed the significance and diversity of contextual factors [12]. In these two studies, community wealth factors were only significant in Burkina Faso, Egypt, and Mali (neither of these studies includes Mozambique).

Although the association between wealth and contraceptive behavior in Mozambique has never been explicitly addressed in detail, contraception has been analyzed from different perspectives. In fact, the contraceptive prevalence for married and in-union women in Mozambique is very low (about 12%) but with striking differences associated with the poverty-wealth dimension: it ranges from 3% to 30% between the first and the fifth wealth quintiles [13]. An analysis of fertility trends based on the first Mozambican Demographic and Health Survey found higher contraceptive prevalence in urban areas and the southern region, where the capital—Maputo—is located, than in the rest of the country [14].

The influence of a woman’s religion on contraceptive behavior has also been studied. Women with Catholic and Protestant affiliations tend to have a higher propensity to use modern contraception than Zionist women and those with other faiths [15]. This association between mainstream religious affiliations and higher contraceptive use has been interpreted as a consequence of the greater social diversity within these congregations that facilitates the social diffusion of new behaviors. Men's migration is another factor influencing contraceptive use, but its effect depends on the economic success of the husband’s migration. Overall, a migrant’s wife tends to adopt modern contraception less frequently; however, women married to economically successful migrants are more likely to do so [16].

Although previous studies have analyzed the effect of distinct socioeconomic factors on contraceptive adoption in Mozambique, none of them have made a comprehensive study of the impact of the poverty-wealth dimension, namely the individual and contextual effects of wealth on contraceptive use.

This paper focuses on the relation between wealth and women's contraceptive behavior in Mozambique using recent data. It applies a multilevel probit regression in which the wealth impact is unmixed into average-community wealth and the differences between the average-community wealth and the original individual score in the wealth index. This separation highlights two distinct pathways for the impact of wealth on the adoption of modern family planning methods: that of the average wealth within the community, and on the other hand, that of the difference between the individual’s wealth and the average wealth of the community. The usual method that combines the average community characteristics with the original individual values does not allow these distinctions to emerge. Our purpose is to disentangle the effects on reproductive health in Mozambique of social diffusion associated with a community's socioeconomic diversity from the individual effects linked to the poverty-wealth dimension.

Regarding the contextual effects arising from the community characteristics—such as the average wealth index—we posit that the greater socioeconomic heterogeneity in the richest communities favors the social diffusion of contraception. This hypothesis is based on Agadjanian’s [15] previous research on Mozambique that shows that the social heterogeneity associated with mainstream religious affiliations, but absent in alternative faiths, was the key factor favoring contraceptive diffusion. Agadjanian’s hypothesis was mainly built on the theory of diffusion [17] that associates diffusion and heterogeneity and on the idea about the influence of weak ties [18]. Gradually, diffusion and social interaction became major topics in subsequent research on declining fertility and contraceptive adoption. Examples include the influential analysis on the interaction processes and channels in contemporary fertility transitions [19], and the question about the roles of social learning and social influence [20] in the diffusion process that marked subsequent research; to sum up, social diffusion of contraception has become a key research question in fertility decline [21].

Following the association between the diffusion of contraception and social heterogeneity, we posit that socioeconomic diversity grows as the average wealth of the community increases, and this social heterogeneity favors the contacts between users and non-users of modern contraception. The stronger presence of women using contraception in the richest communities increases the odds of non-users coming into contact with it. Consequently, it improves the exposure to non-formal channels of information and the adoption of new role models.

Not only is the diffusion process facilitated in the richest communities, but they also tend to live in healthier environments, e.g. access to clean water and electricity and particularly to more and better health facilities. This is a key point since the presence of health facilities and their features impacts women's contraceptive choices [22].

Social interaction has two main channels: geographical proximity and social proximity [19]. If contextual effects are associated with the local residence, the social proximity depends on individual characteristics.

Looking at the effects of the individual's position within the local community, we posit that contraceptive adoption is associated to the women's socioeconomic position in each community. Women from the richest households are more likely to belong to, or at least to be associated to, the local elites and establish ties with other community elites. Consequently, they tend to have more contact with new information, attitudes, role models and behaviors concerning day-to-day life, healthcare and, in this case, the adoption of modern family planning methods. As the women's position in the local hierarchy increases, all these factors raise the odds of using contraception.

To sum up, not only does the socioeconomic heterogeneity allow the diffusion of information and new attitudes but also the proximity with the forerunners favors that diffusion and change in contraceptive behavior.

The next section describes the data sample, variables, and methods. The sample's dependent and independent variables are then presented and the results for the single level and multilevel models are reported. The paper ends with a discussion of the implications of these results.

## Data and Methods

### Sample

The data set used in the empirical analysis comes from the 2011 Mozambique Demographic and Health Survey (DHS) [13], retrieved from MEASURE DHS public repository (site: dhs.program.com). The data set was provided for research use in an anonymized version and hence no formal ethical approval was necessary. This dataset has information on both wealth and contraceptive use; furthermore, it has relevant information on other individual and household factors discussed in the literature: region, residence, religion, age, marital situation, previous fertility, work, and education. The original data file contains 13745 women aged from 15 to 49 years. However, this research reduced the original sample to 7530 observations with the aim of focusing only on married women or those living in marital union, not pregnant or sterilized and aged between 15 and 49 years. The sample also excludes women who do not usually live in the household as the wealth index is measured at household level. Moreover, given the intention to address the community poverty-wealth effect on contraceptive use, we excluded communities represented by less than 6 women in the non-weighted sample to avoid extreme imbalance in the number of observations within each level (the sample loss with 5 observations is about 0.3% for both urban and rural samples; the loss is 1.9% and 0.4% with 6 observations; 3.4% and 0.9% with 7 cases; and 8.0% and 1.9% with 8 observations—the minimum of 6 observations ensures enough variability without strong bias in the sample). Tabulations and econometric models were computed on the weighed database using the weights provided in the sample.

### Variables

As the purpose of this analysis is to disentangle the wealth effects on contraceptive adoption, the dependent variable is the current use of modern contraception. It is dichotomous and the ‘yes’ encompasses users of any kind of modern birth control (injections, pill, condom, lactational amenorrhea, female sterilization, IUD or other modern method); all other situations are included in the ‘no’ answer (periodic abstinence, withdrawal or other, as well as the non-use of family planning methods).

The independent variables are defined at individual/household and community levels: life-cycle factors (e.g., woman's age and previous fertility) as well as marriage characteristics (married vs. living in union, the existence of other wives and the husband's residence in the home), woman's education, working situation, religious affiliation, place of residence (rural or urban), region, the woman's opinion on the distance to get medical care, and wealth index. The wealth index is a composite variable based on a principal component analysis of household assets and housing characteristics; therefore, it is a standardized variable [23] and included in the DHS 2011 data set.

The community level analysis includes three explanatory variables—the average wealth index of the community, place of residence, and region—as they do not vary at the primary sampling unit (PSU) level. It is important to stress that most of the variation in the wealth index comes from the differences between the averages of the community of residence (Eta squared = 0.83). This feature highlights the need to address the relationship between contraception and wealth with a multilevel framework.

The unmixing will reveal two components of the wealth impact: the contextual effect incorporated in the community's average wealth and the effect of the woman's position within a specific community addressed by the wealth-difference variable.

### Methods

The hierarchical structure of the DHS data violates the assumption of independence as women are clustered within communities of residence (primary sampling units—PSUs). Hence, the dependent variable—current use of modern contraception—is analyzed using a multilevel model that takes the interactions between the two levels of the hierarchy into account [24–25]. Additionally, standard errors are not underestimated as they do not assume the independence of observations.

Let *j* and *i* denote level-2 (cluster or community) and level-1 (nested observations within each community), respectively. The total number of level-2 units is indicated by *N* and within cluster *j* is designated by *n*_*j*_. Total sample size (level-1) is $n=\sum_{j=1}^{N}n_{j}$. The dependent variable for level-1 unit *i* in level-2 cluster *j* is *Y*_*ij*_ and is coded as either 1 (success: use of modern contraception) or 0 (failure: no use of modern contraception). Let *p*_*ij*_ be the probability of success for *i* (women) in cluster *j*, i.e., *p*_*ij*_ = *P*(*Y*_*ij*_ = 1). The community or place of residence constitutes the second level in the multilevel models taking into account the hierarchical structure of data and adjusting for the community effects. It is given by the Primary Sample Unit (PSU) in the DHS. Additionally, if the latent variable $Y_{ij}^{*}$ is above a given threshold *τ*, then we observe a success, i.e., $Y_{ij}=I(Y_{ij}^{*}>\tau )$, where *I*(.) is the indicator function: *I*(*true*) = 1; and 0, otherwise. The linear component of the model with a random effect is given by $Y_{ij}^{*}=x_{ij}^{'}\beta +u_{j}+\epsilon_{ij}$, in which ***x***_*ij*_ is the vector that contains the covariates for observation *i* in cluster *j*, ***β*** is the vector of regression parameters (fixed effects), *u*_*j*_ is the random effect for cluster *j*, and *ϵ*_*ij*_ is the error term. The random effect ($u_{j}$) represents unobserved level-2 factors affecting $Y_{ij}^{*}$ that are shared by all units within cluster *j* after controlling level-1 and level-2 covariates. This model estimates the threshold parameter as opposed to an intercept parameter. The probit regression framework assumes standard normal errors, i.e., *ϵ*_*ij*_ ∼ *N*(0,1). Thus, the probability of success using the probit-link function is $p_{ij}\;=\;\Phi (-\tau +x_{ij}^{'}\beta +u_{j})$, where Φ(.) is the distribution function of the standard normal distribution. The random intercepts *u*_j_ are independent of the errors (*ϵ*_*ij*_) and normally distributed: $u_{j}\sim N(0,\sigma_{u}^{2})$. The intraclass correlation (ICC) corresponds to the proportion of the total variability that is explained by the level-2 clustering and is given by $\mathrm{ICC}=\sigma_{u}^{2}/(1+\sigma_{u}^{2})$.

Based on this multilevel probit regression framework, we set up four models. Models 1 and 2 do not assume a clustering effect of the community at the upper level, relaxing the multilevel structure ($\sigma_{u}^{2}=0$ and, thus, *u*_*j*_ = 0). Model 1 includes the key variable—wealth index—, whereas Model 2 includes all important variables at the individual level. Model 3 adds a random intercept to Model 2, which measures unobserved effects at community level.

Since the DHS does not collect community level data, we compute the community level wealth index by averaging individual level data at the PSU level. Wealth group-mean centering was applied. It has been shown that group-mean centering provides both a meaningful zero point for predictors and unbiased estimates of individual level slopes, which is not always the case with grand-mean centering [26–27]. Thus, Model 4 unmixes the effect of wealth into individual-level effect (difference between household wealth and community wealth) and community-level effect. This effect is given by $\beta_{Wealth}^{\left(1\right)}({Wealth}_{ij}-{Wealth}_{j})+\beta_{Wealth}^{\left(2\right)}{Wealth}_{j}$, where $\beta_{Wealth}^{\left(1\right)}$ and $\beta_{Wealth}^{\left(2\right)}$ are the effect of the wealth differences within the woman’s own community of residence and average community wealth, respectively. Notice that the wealth of the community is the average wealth of all households in that community, i.e., $Wealth_{j}=n_{j}^{-1}\sum_{i=1}^{n_{j}}Wealth_{ij}$. This separation will reveal two components of the wealth impact: the contextual effect of the community average wealth and the effect of the individual position within a specific community addressed by the wealth-difference variable. The individual difference vis-à-vis the average wealth of their community denotes each woman's position in the economic pyramid within their place of residence, rather than a comparative measure between communities. Thus, at community level Model 4 includes three independent variables: the average wealth index of the community, place of residence, and region. We run Model 4 for urban and rural regions separately.

## Results

Table 1 highlights the key characteristics of the sample. The large majority of the women live in rural areas, where the prevalence rate of modern contraception is considerably lower than in urban areas. The geographical differences are also quite substantial: the use of modern contraception in some areas is very low (Cabo Delgado, Nampula, and Zambézia), whereas in other regions it is high (Gaza and Tete). Maputo region, where the capital city is located, has the highest prevalence rate and its surrounding area is also characterized by high modern family planning adoption. Women’s religious affiliation reveals the stark difference in the prevalence rate of Islamic women, which is clearly lower than all other religious affiliations. On the other hand, women with Evangelical/Pentecostal and Zion religious affiliations show the highest prevalence in the use of modern contraceptives.

**Table 1:** **Sample description and modern contraceptive prevalence (Mozambique DHS 2011)**

	Unweighted N	Weighted %	Modern Contraception %
**Type of residence**			
Urban	2686	29.6	23.2
Rural	4844	70.4	8.5
**Region**			
Niassa	577	5.5	13.6
Cabo Delgado	678	8.5	3.2
Nampula	563	14.4	6.2
Zambezia	809	19.6	5.0
Tete	663	12.2	17.7
Manica	709	7.2	14.6
Sofala	872	9.9	8.8
Inhambane	645	6.3	13.1
Gaza	659	5.5	19.8
Maputo	1355	11.0	36.2
**Religion**			
Catholic	1851	28.6	12.8
Evangelical/Pentecostal	1398	17.1	16.7
Zionist	1708	18.4	15.5
Islamic	1204	18.3	7.4
Other	1369	17.7	12.2
**Age**			
15–19 years	796	10.8	7.5
20–24 years	1375	18.2	14.2
25–29 years	1468	19.1	16.5
30–34 years	1289	17.0	15.7
35–39 years	1135	15.1	14.1
40–44 years	795	10.6	9.1
45–49 years	672	9.2	6.1
**Marital Status**			
Married	4485	65.3	10.7
Living with partner	3045	34.7	16.9
**Currently residing with husband/partner**			
Yes	6258	85.7	12.9
No	1272	14.3	12.6
**Other wives**			
No	5450	73.9	12.6
Yes	2080	26.1	13.8
**Living Children**			
0	721	9.4	1.8
1	1394	18.4	13.1
2	1347	17.5	15.9
3	1229	15.5	14.7
4+	2839	39.1	13.4
**Distance to medical help**			
Big problem	3780	56.7	8.0
Not a big problem	3750	43.3	19.2
**Education**			
No education	2496	36.3	6.1
Primary	3892	51.7	12.7
Secondary + Higher	1142	11.9	34.1
**Female occupation**			
No	3819	49.8	13.8
Yes	3711	50.2	12.0
**Wealth Index**			
Poorest	1076	19.3	3.6
Poorer	1310	20.5	6.5
Middle	1482	20.9	8.0
Richer	1681	20.5	15.7
Richest	1981	18.7	31.7

Note: Number of respondents is based on unweighted data; percentages are sample weighted-adjusted.

The age factor takes an inverted U-shaped pattern for contraception; this is probably due to the smaller number of offspring at younger ages and, on the other hand, to the fecundity of the oldest women being the lowest (as in [13]). Both situations reduce the need to use family planning methods. Major differences are found for contraceptive use in relation to the number of living children: it is almost nonexistent for childless women but rather homogeneous for mothers irrespective of the number of living children. Women's marital circumstances are also important: contraceptive prevalence is lower for married women than for those living with a partner; there is also a small difference between the women in a monogamous and polygamous union and depending on whether or not she lives with her partner. Working women show lower contraceptive adoption. Women's opinions on the distance to medical assistance reflect major differences in contraceptive prevalence: when the distance is considered a big problem, contraceptive adoption is considerably lower. The socioeconomic gradients are observed in education and wealth—these two variables reveal the most substantial differences in contraceptive prevalence (similar to those associated to the geographical context). The use of contraception rises as women's education and wealth increase. To sum up, the major differences in contraception adoption are associated with residence and socioeconomic gradients. This underlines the need to address the poverty-wealth impact on contraceptive use within a contextual framework supported by a multilevel model.

Table 2 reports results for four probit regression models. The first two models are single level, whereas the last two have a multilevel structure. Model 1 only includes the wealth index, which reveals a significant linear effect. Model 2 controls for other individual factors that reduce the impact of the wealth index on the use of contraception. Region, religion, education, age, living in the same household as the partner and the number of living children have a significant impact on the use of modern contraceptive methods. The most important effects are associated with region, age, and the number of offspring. As for the socioeconomic factors, women's education and household wealth have significant positive effects on the adoption of modern family planning methods.

**Table 2:** **Probit regression models for contraceptive use (ref: non-users).**

	Model 1		Model 2		Model 3		Model 4	
Level 1—Women								
Wealth index								
Linear	0.464	***	0.205	***	0.214	***	-	
Difference	-		-		-		0.180	***
Education (Ref: No formal education)								
Primary	-		0.280	***	0.300	***	0.298	***
Secondary or Higher	-		0.658	***	0.732	***	0.725	***
Religion (ref: Catholic)								
Evangelical/Pentecostal	-		-0.104		-0.043		-0.041	
Zionist	-		-0.157	*	-0.103		-0.103	
Islamic	-		-0.048		-0.026		-0.032	
Other	-		-0.260	**	-0.250	***	-0.251	***
Age (ref: 20–24)								
15–19 years	-		-0.090		-0.091		-0.092	
25–29 years	-		-0.027		-0.064		-0.064	
30–34 years	-		-0.142		-0.210	**	-0.212	**
35–39 years	-		-0.197	*	-0.284	***	-0.287	***
40–44 years	-		-0.482	***	-0.584	***	-0.588	***
45–49 years	-		-0.695	***	-0.876	***	-0.880	***
Marital status (ref: Married)								
Other	-		-0.059		-0.052		-0.054	
Currently residing with husband/partner (ref: yes)								
No	-		-0.139	*	-0.199	**	-0.200	**
Other wives (ref: No)								
Yes	-		0.042		0.046		0.047	
Female occupation (ref: Not working)								
Working	-		0.003		0.058		0.059	
Distance to medical help (ref: no big problem)								
A big problem	-		-0.080		-0.034		-0.028	
Living children (reference: 1)								
0	-		-0.965	***	-0.910	**	-0.908	***
2	-		0.195	*	0.277	***	0.275	***
3	-		0.207	*	0.346	**	0.346	***
4+	-		0.467	***	0.639	***	0.642	***
Thresholds	1.156	***	1.666	***	1.872	***	1.837	***
Level 2—Communities								
Wealth index								
Wealth Index (PSU)	-		-		-		0.260	***
Residence (ref: Rural)								
Urban	-		0.123		0.130	*	0.084	
Region (ref:Nampula)								
Niassa	-		0.384	**	0.417	**	0.416	**
Cabo Delgado	-		-0.288		-0.284		-0.289	
Nampula	-		-0.117		-0.153		-0.154	
Tete	-		0.710	**	0.711	***	0.696	***
Manica	-		0.362	**	0.316	*	0.298	*
Sofala	-		0.143		0.204		0.191	
Inhambane	-		0.415	**	0.400	**	0.373	**
Gaza	-		0.693	**	0.701	***	0.669	***
Maputo	-		0.724	***	0.751	***	0.694	***
Var(*u*_*j*_)	-		-		0.065	***	0.065	***
ICC					0.061		0.061	

Notes: Residual variance is equal to 1;

*** (p < 0.001),

** (p < 0.01),

* (p < 0.05).

Model 3 includes the woman's residence in a particular community (PSU) as the second level of analysis. This inclusion accounts for 6.1% of the total variance (ICC). The model confirms the poverty-wealth effects on contraceptive use: the impact is significant, positive and about the same size as in the single level model. But the residence variable has a significant effect in this model: women from urban settings are more likely to adopt contraception. The inclusion of the higher level in the model also eliminates the negative effect of the Zion religious affiliation. As before, women's marital characteristics do not impact contraceptive use but the co-residence with the partner does. There are no effects arising from the women's occupations. The women's opinion about the distance to medical help does not impact contraceptive use. There is a clear gradient associated with education: as education increases, so does the use of contraception. This educational impact on modern contraceptive use is consistent with the poverty-wealth impact, as expected.

Finally, Model 4 redefines the poverty-wealth dimension using group-centering. The household wealth score is unmixed into two components: the community's average wealth (mean wealth index from PSU) and the difference between the individual score and this average (wealth index difference). The model confirms the effects found in the previous model for all other explanatory variables. The exception is that, in this model, the urban rural differences do not impact the probability of women adopting contraception. Regarding the wealth index, the results for the two variables are clear. The community's average wealth has a significant positive impact on contraceptive use and the same is true for the individual position within the community. The impact of the community's average wealth on contraceptive use is higher than that of the individual difference.

Additionally, a consistency check was performed on the wealth effects. In order to understand if the contextual effects of the wealth factor are dependent on the community educational levels, we run an alternative model where both wealth and education were decomposed into the community averages and the individual differences to those local averages (results not shown). After controlling for contextual education, the effects from the community wealth and the individual wealth differences remain positive and significant.

Research has shown that the wealth level is substantially different in rural and urban areas. The dissimilarity is so marked that in some studies the wealth index is computed separately for urban and rural regions [28–29]. In contrast, the option in this research was to model urban and rural areas separately with the same wealth index in order to preserve comparability of the wealth effects between rural and urban settings (Table 3).

**Table 3:** Probit regression models for contraceptive use in urban and rural areas (ref: non-users)

	Model 4 -	Urban	Model 4 -	Rural
** Level 1—Women**				
**Wealth index**				
**Linear**	-		-	
Difference	0.184	**	0.188	*
**Education (Ref: No formal education)**	** **	** **	** **	** **
Primary	0.338	**	0.300	***
Secondary or Higher	0.745	***	0.686	***
**Religion (ref: Catholic)**	** **	** **	** **	** **
Evangelical/Pentecostal	-0.134		0.031	
Zionist	-0.291	**	0.057	
Islamic	-0.001		-0.101	
Other	-0.152		-0.349	**
**Age (ref: 20–24)**	** **	** **	** **	** **
15–19 years	-0.016		-0.214	
25–29 years	-0.117		-0.007	
30–34 years	-0.271	**	-0.171	
35–39 years	-0.490	***	-0.122	
40–44 years	-0.824	***	-0.390	**
45–49 years	-1.248	***	-0.529	***
**Marital status (ref: Married)**	** **	** **	** **	** **
Other	-0.053		-0.053	
**Currently residing with husband/partner (ref: yes)**	** **	** **	** **	** **
No	-0.319	***	-0.106	
**Other wives (ref: No)**	** **	** **	** **	** **
Yes	0.046		0.029	
**Female occupation (ref: Not working)**	** **	** **	** **	** **
Working	0.102		0.043	
**Distance to medical help (ref: no big problem)**	** **	** **	** **	** **
A big problem	-0.018		-0.029	
**Living children (reference: 1)**	** **	** **	** **	** **
0	-0.868	***	-0.916	***
2	0.382	***	0.139	
3	0.525	***	0.149	
4+	0.832	***	0.448	***
**Thresholds**	1.373	***	1.772	***
** Level 2—Communities **				
**Wealth index**				
Wealth Index (PSU)	0.259	***	0.475	***
**Residence (ref: Rural)**				
Urban	-		-	
**Region (ref:Nampula)**				
Niassa	0.061		0.564	**
Cabo Delgado	-0.543	*	-0.246	
Nampula	-0.434		-0.165	
Tete	-0.229		0.908	***
Manica	-0.077		0.268	
Sofala	-0.368		0.357	*
Inhambane	-0.107		0.371	*
Gaza	0.466	*	0.491	**
Maputo	0.330		0.535	*
**Var(*u***_***j***_ **)**	0.001		0.102	***
**ICC**	0.001		0.093	

Notes: Residual variance is equal to 1;

*** (p < 0.001),

** (p < 0.01),

* (p < 0.05).

In urban areas the community of residence is not an important feature (ICC = 0.001), that is, the individual observations are almost independent of each other and the multilevel structure does not prove to be necessary. In fact, in urban areas the definition of the primary sampling units, on which the multilevel analysis is built, does not match the women's social interaction boundaries.

The impact of the individual level variables is significant for the same factors, but there are marked differences. For instance, women with Zion affiliation are less likely to adopt contraception (as in [15]); the age gradient is more pronounced than in the overall model; and the negative impact of the partner's non-residence is also stronger. Our main variable—wealth—reveals similar effects to the ones found previously in model 4 for the whole country.

In rural areas, the community of residence is an important characteristic that must be included in the models (ICC = 0.093). When compared to urban areas, rural areas have greater proximity between the geographical limits of the residence (primary sampling units) and the boundaries associated with individuals’ social interaction. The magnitude of the gradients for women's age and the number of offspring is smaller in rural than urban areas. There is no effect from the partner's residence; on the other hand, religious affiliation only affects contraception for women in the “other” category. Women's education has quite a similar effect in rural and urban contexts. Finally, the wealth factor: the impact of women's position in the socioeconomic pyramid is equivalent to that of urban areas, but the community's average wealth has a much greater impact on contraceptive adoption than in the urban area.

The strong impact of the community's average wealth on contraceptive adoption in rural areas is a key point to our hypothesis about social diffusion. In order to understand these effects, we classify the communities of residence into quintiles in accordance with the PSU average wealth. Table 4 shows the dispersion of wealth index measures at community and individual levels using quintiles.

**Table 4:** **Standard deviation of wealth measures**

	All Sample		Urban Sample		Rural Sample	
**Quintiles (based on WI-PSU)**	**WI**	**WI-Diff**	**WI**	**WI-Diff**	**WI**	**WI-Diff**
**1**	0.101	0.096	0.397	0.333	0.083	0.080
**2**	0.165	0.163	0.615	0.576	0.146	0.145
**3**	0.316	0.304	0.748	0.716	0.179	0.177
**4**	0.571	0.528	0.512	0.497	0.343	0.332
**5**	0.739	0.552	0.364	0.320	0.727	0.542

In country-level and rural-level analyses, the more wealth a community has, the more its socioeconomic heterogeneity grows. The increase in the standard deviation is crucial: for the whole country and in rural areas, the increase in the community's average wealth accompanies the growing socioeconomic diversity within each community. This finding supports the first hypothesis posited for the wealth-contraception association, i.e., there is greater socioeconomic heterogeneity in the richest communities and this favors the social diffusion of contraceptive behavior. In contrast, the dispersion in the urban setting reveals an inverted U-shaped curve, so we can expect that social diffusion is higher in the central quintiles.

As for the wealth index, the wealth difference—the variable that measures the individual position within the community—shows a similar pattern to the original wealth score. As the community average wealth increases, so does the internal variability. This shows that unmixing the average community wealth from the individual difference preserves the differentiation between individuals associated with the local social stratification within the community. It also shows that the within community diversity is present even in the poorest communities and this must be addressed in the analysis of the wealth-contraception relationship in order to analyze the second hypothesis posited for the wealth-contraception relationship: the effect of the women's position in the local socioeconomic ranking on the adoption of family planning methods. It should be noted that as most of the sample is rural (70%), the results for these areas show strong similarities to those of the whole country.

## Conclusion

Mozambique is an eye-catching case since it is a particularly poor country with low contraceptive prevalence and high fertility levels. This analysis highlights the need for the studies on contraceptive behavior to make a distinction between the individual and contextual effects arising from the poverty-wealth dimension and separated in rural and urban areas. In short, these results confirm that the positive association in Mozambique between individual and community wealth remains significant after controlling other important covariates.

The unmixing of the rural and urban areas reveals very marked differences. Whereas the inclusion in a particular community of residence is not relevant in urban areas, it is an important feature in rural areas. Additionally, although the effect of the women's individual position within the community of residence on contraceptive adoption is similar in rural and urban settings, the impact of community wealth is greater in rural areas and smaller in urban areas.

Most of the population live in rural areas and this is where we find a positive association between the average wealth of the community of residence and the wealth variability within the community. The greater socioeconomic heterogeneity in the richest communities favors the vertical diffusion of new behaviors regarding modern contraception. In contrast, the diffusion of new behaviors is less likely in the poor and more homogeneous communities since non-users have less contact with women already using contraception.

The analysis of contextual effects grounded on the community averages and the individual differences to the community average is technically more accurate than the usual approach based on the original individual scores (without differentiation) together with the community mean. Models using this specification show that, in Mozambique, the individual differentiation within the community of residence impacts women's behavior towards contraception similarly in rural and urban areas. Contraceptive behavior is influenced by contextual effects associated with the average community wealth. Adopting modern family planning methods proves to be more difficult in the poorest communities, particularly if they are rural. These findings are relevant for family planning policies, which should be tailored to the specific features of the local communities to maximize their effectiveness.

To sum up, the wealth effect must be considered both at individual and contextual levels. In an attempt to frame these two effects, we posed two hypotheses on the social diversity and the individual proximity to the forerunners. These hypotheses were explored using the multilevel decomposition of the wealth effect. A more detailed analysis of these hypotheses would require data on the social networks of Mozambican women, which are not available in the DHS data. In fact, these detailed data on the dimension, diversity and socioeconomic characteristics of women's networks, along with their contraceptive practices, are necessary to explore the causal mechanism. Thus, intensive rather than extensive studies would be a fruitful complement to our country-level analyses.
